# Effects of mTOR inhibitors and cytoskeletal-directed agents alone and in combination against normal and neoplastic hematopoietic cells in vitro

**DOI:** 10.1007/s10637-015-0294-7

**Published:** 2015-10-22

**Authors:** Matthew Trendowski, Timothy D. Christen, Antoaneta A. Andonova, Berlini Narampanawe, Ashlee Thibaud, Tenzin Kusang, Thomas P. Fondy

**Affiliations:** Department of Biology, Syracuse University, 107 College Place, Syracuse, NY 13244 USA

**Keywords:** Leukemia, Hematopoietic stem cells, MTOR inhibitors, Cytoskeletal-directed agents, Drug synergy

## Abstract

The mechanistic target of rapamycin (mTOR) controls cell growth and enlargement and has been found to be aberrant in a wide variety of malignancies. Although mTOR is already an attractive antineoplastic target, overexpression or aberrant expression of mTOR may also provide an opportunity to further increase the size differential between malignant and normal cells, providing an opportunity to amplify and exploit cell size differences between neoplastic cells and their normal counterparts using physiochemical treatment modalities. Therefore, this study sought to quantify the concentration response and time course effects of rapamycin on cell cycle entry, cell enlargement, and cell proliferation in U937 human monocytic leukemia and human hematopoietic stem cells (hHSCs). In addition, the effects of combination treatment with mTOR inhibitors (rapamycin, everolimus, and temsirolimus) and cytoskeletal-directed agents (cytochalasin B and vincristine) in leukemic cells (U937, THP1, K562, Molt-4, and L1210) were assessed for potential drug synergy. While both U937 cells and hHSCs exhibited a marked reduction in cell volume, U937 cells were able to proliferate in the presence of rapamycin ranging from 0.5 nM to 10 μM (10,000 nM), whereas hHSCs were able to proliferate only at lower concentrations, and were completely inhibited from proliferation by 8 nM rapamycin. These effects were observed with as little as 0.5 nM rapamycin, demonstrating the profound affinity the compound has for FK-binding protein 12 (FKBP12), which subsequently forms the FKBP12/rapamycin complex to inhibit mTOR. Rapamycin continued to exert effects on cell size and proliferation even at 10 μM, without producing marked cytotoxicity. Although cytochalasin B and vincristine were unable to substantially enlarge rapamycin-treated leukemia cells, it appears that rapamycin and its associated analogs everolimus and temsirolimus have notable synergistic potential with microfilament-disrupting cytochalasin B and microtubule-disrupting vincristine as assessed by comparative effects on cell growth, annexin V staining, IC_30_ isobolograms, and Chou-Talalay statistics. These observations indicate a potentially novel therapeutic rationale for hematological malignancies and for other cancers to elicit the preferential destruction of neoplastic cells that aberrantly express mTOR.

## Introduction

The mechanistic target of rapamycin (mTOR) has received considerable attention as a potential target for cancer chemotherapy. As a member of the phosphatidylinositol 3-kinase-related kinase protein (PIKK) family, mTOR is a serine/threonine protein kinase that regulates cell growth, cell proliferation, cell motility, cell survival, protein synthesis, and transcription, and is a constituent of the PI3K/AKT/mTOR (PI3K: phosphatidylinositol-4,5-bisphosphate 3-kinase; AKT: protein kinase B) pathway [1]. Due to its substantial regulatory control on normal cellular function, mutations involving the mTOR pathway considerably increase the likelihood of carcinogenesis. Indeed, aberrant mTOR regulation has been found in a considerable diversity of cancer types, including breast, lung, and pancreatic carcinomas, as well as hematological malignancies [2–5]. These studies have confirmed that mTOR is indeed a commonly aberrant pathway constituent in malignant cells, contributing substantially to the pathogenesis of many malignancies. As such, virtually all research involving the mTOR pathway has been directed toward inhibiting mTOR as a key oncoprotein. This is to be expected, as oncogenes and their associated oncoproteins facilitate cancer pathology through increasing growth and proliferation, as well as establishing an immortalized cellular phenotype [6].

However, there are other approaches to obtaining preferential damage against malignant cells. It is well known that malignant cells have a perturbed cytoskeleton due to the effects of dysplasia and subsequent anaplasia [6]. With so many alterations present in malignant cells, the cytoskeleton provides an ideal opportunity to obtain preferential damage through a physical modality. While neoplastic cells have a considerable propensity to acquire drug resistance to persistent chemotherapeutic exposure, it is very difficult for cytoskeletal integrity to be reinforced after exposure to a physical disturbance [6, 7]. In an approach known as sonodynamic therapy (SDT), specialized chemotherapeutic agents known as sonosensitizers are administered to increase the extent of preferential damage elicited by ultrasound against neoplastic cells. It has been repeatedly shown that ultrasound preferentially damages malignant cells based on the size differential between such cells and those of normal histology [7–12]. This known fact suggests that enlarging neoplastic cells to increase their already noticeable size difference with normal cells could be a method by which to attain further preferential damage.

Since overexpression of mTOR has been extensively associated with increased proliferation rates in hematological malignancies, exploiting the aberrant pathway may be a potential approach to further increase the size differential between normal and neoplastic cells. mTOR acquired its name due to its profound sensitivity to rapamycin, a bacterial toxin produced by *Streptomyces hygroscopicus* [13, 14]. Although the true binding target of rapamycin is FK-binding protein 12 (FKBP12), the FKBP12/rapamycin complex potently inhibits the function of mTORC1, and to a certain extent mTORC2. Initially, rapamycin (sirolimus) was employed as an immunosuppressive drug following organ transplantation, as it suppresses mammalian immune systems by blocking the G_1_ to S phase transition in T-lymphocytes [13, 14]. Therefore, rapamycin inhibition of mTOR prevents normal immune-response cells from completing mitosis by preventing cell cycle progression. Since its introduction as an immunosuppressive agent, the antineoplastic activity of rapamycin has been widely noted, and its derivatives everolimus and temsirolimus are used in the clinical setting for the treatment of localized solid tumors, as well as disseminated cancers [1–4]. However, it may be the case that leukemias and other hematological malignancies have acquired enough mutations to become resistant to rapamycin exposure. As such, the malignant cells would continue through the cell cycle and complete mitosis, thereby amplifying the already substantial size difference between leukemic and normal blood cells. Further, it is likely that cell enlarging microfilament- and microtubule-directed agents that severely perturb mitosis could considerably amplify this size difference, potentially enhancing the efficacy of these agents.

Exploiting aberrant mTOR signaling in leukemias and other hematological malignancies may indeed provide a reliable basis to preferentially enlarge malignant cells under physiological conditions. Such size differences may be exploited by physicochemical therapeutic approaches that specifically target large cells with weakened cytoskeletal integrity. Therefore, this study seeks to compare the physiological responses of malignant and normal blood cells after exposure to rapamycin. In addition, normal and neoplastic hematopoietic cells are treated with cell enlarging cytoskeletal-directed agents (cytochalasin B and vincristine) alone and in combination with mTOR inhibitors (rapamycin, everolimus and temsirolimus) to determine whether marked preferential enlargement and damage of leukemic cells can be attained.

## Materials and methods

### Preparation of leukemia cell lines and normal blood cells

U937 human monocytic leukemia cells (ATCC® CRL-1593.2) were placed at 5.2 × 10^4^ viable cells/ml in 20 % fetal bovine serum (FBS) in Iscove’s medium without glutamine, with the following added: 200 units/ml penicillin, 200 μg/ml streptomycin, 100 μg/ml gentamicin sulfate, 40 μM glutamine (50 μl of 2 mM glutamine per 5 ml medium), and 50 μl of amphotericin B (2.5 μg/ml concentration) per 5 ml of medium. K562, Molt-4, and THP1 human leukemia (ATCC® CCL-243, CRL-1582, TIB-202), as well as L1210 murine leukemia (ATCC® CCL-219) were cultured under the same conditions. Human hematopoietic stem cells (hHSCs) acquired from the State University of New York Upstate Medical University (Syracuse, NY, USA) were cultured under the same conditions after their use was approved by an IRB protocol. Cells were incubated in 5 % CO_2_ in a humidified chamber at 37 °C. Viability was assessed by 0.4 % trypan blue stain in isotonic saline, followed by cell counting and sizing using a Z2 Beckman-Coulter® Particle Count and Size Analyzer (Beckman Coulter Inc., Brea, CA, USA), along with a Bio-Rad® TC20 Automated Cell Counter (Bio-Rad Laboratories, Inc., Hercules, CA, USA). Extent of multinucleation after treatment with rapamycin or cytoskeletal-directed agents was assessed with Wright stain.

### MTOR inhibitor preparation and administration

Rapamycin (Sigma-Aldrich Corp., St. Louis, MO, USA) was prepared in 40 μM stock solutions using 95 % EtOH (Sigma-Aldrich Corp.). Rapamycin analogs everolimus and temsirolimus were prepared using the same conditions. Cell size, viability, and proliferation rates were determined by the cell counters. Vehicle controls of 95 % EtOH were tested in parallel with the rapamycin-treated cells.

### Effects of cytoskeletal-directed agents on rapamycin activity

8 nM vincristine was administered for an additional 25 h after cells were exposed to 10 μM rapamycin for 24 h. The 10 μM rapamycin was removed prior to vincristine administration, but due to the high concentration of rapamycin prior to removal, a residual concentration of 40 nM rapamycin was left in solution, along with the 8 nM vincristine. Vincristine-treated U937 cells were then compared to controls that received only rapamycin to determine whether cell size effects of rapamycin could be affected by the microtubule-altering agent in neoplastic or normal cells. 4 nM vincristine and 2 μM cytochalsin B were similarly tested with a 12 h and 48 h exposure, respectively after treatment with 50 nM rapamycin for 24 h.

### Effects of concomitant administration of mTOR inhibitors and cytoskeletal-directed agents against normal and neoplastic hematopoietic cells

To assess the cytotoxic effects of rapamycin and two of its associated analogs (everolimus and temsirolimus; Sigma-Aldrich Corp.) with cytochalasin B and vincristine, multiple leukemia cell lines (U937, THP1, K562, Molt-4, and L1210 were exposed to varying concentrations of the abovementioned mTOR inhibitors and cytoskeletal-directed agents, both alone and in combination. Cytotoxicity was assessed by comparative effects on cell growth and annexin V staining (Life Technologies, Grand Island, NY, USA). Potential drug synergy was evaluated with IC_30_ isobolograms (IC_30_ values were chosen due to the fact that rapamycin did not inhibit U937 cell growth beyond approximately 44 %). In addition, the Chou-Talalay method for assessing drug synergism was implemented to determine the combination index (CI) and fraction affected (Fa). As indicated in [15], drug synergy was assessed with the following values: CI < 1 (synergism), CI = 1 (additive), and CI > 1 (antagonism).

## Results

### Effects of 48 hour rapamycin treatment on the cell size of U937 human monocytic leukemia and human hematopoietic stem cells

The effects of rapamycin at concentrations ranging from 0.5 to 32 nM are shown in Table 1. The differential effect of rapamycin on the sizes of normal and leukemic cells was apparent at concentrations at and below 32 nM, with differential inhibition amounting to 2-fold based on cell volume percent reduction (Table 1). Nevertheless, both U937 cells and hHSCs appeared to have a marked reduction in size 48 h after rapamycin administration at 25 nM or higher (Table 2). As seen in Table 2, 25 nM rapamycin inhibited hHSC cell size by 24 %, while U937 cells were reduced by only 12 %, suggesting that the leukemia cells were more resistant to the effects of rapamycin at or below 25 nM. At 25 nM rapamycin, the leukemia cells remained quite large with an average volume of 1829 μm^3^, while hHSCs had an average volume of 698 μm^3^. The effects of rapamycin on cell size were concentration-dependent from 25 nM to 200 nM for U937 cells, but not for hHSCs. Size reduction for U937 cells remained at 30 % at 400 nM and 800 nM rapamycin. For hHSCs, size was reduced by as much as 31 % at 50 nM, with the size inhibition leveling off at higher concentrations. It should be noted that the average cell volume for untreated U937 cells and hHSCs was 15 % larger in Table 2 compared to Table 1 due to different times of cell growth, with the distribution of cells at slightly different places in the cell cycle.

**Table 1:** Effects of rapamycin concentrations ranging from 0.5 to 32 nM on the average cell volume of U937 human monocytic leukemia and human hematopoietic stem cells 48 h post-rapamycin treatment

Rapamycin concentration (nM)	U937 Average volume (μm^3^)	% Size inhibition U937	hHSC Average volume (μm^3^)	% Size inhibition hHSC
0	1800	0	742	0
0.5	1798	0.02	760	−0.24
1	1763	2.1	723	2.6
2	1752	2.7	704	5.2
4	1740	3.4	702	5.4
8	1710	5	668	10
16	1675	7	632	14.8
32	1543	14.3	601	19.1

**Table 2:** Effects of rapamycin concentrations ranging from 25 to 800 nM on the average cell volume of U937 human monocytic leukemia and human hematopoietic stem cells 48 h post-rapamycin treatment

Rapamycin concentration (nM)	U937 Average volume (μm^3^)	% Size inhibition U937	hHSC Average volume (μm^3^)	% Size inhibition hHSC
0	2083	0	900	0
25	1829	12	698	24
50	1536	24	619	31
100	1500	28	682	24
200	1480	29	645	28
400	1458	30	715	21
800	1462	30	672	25

**Fig. 1:**
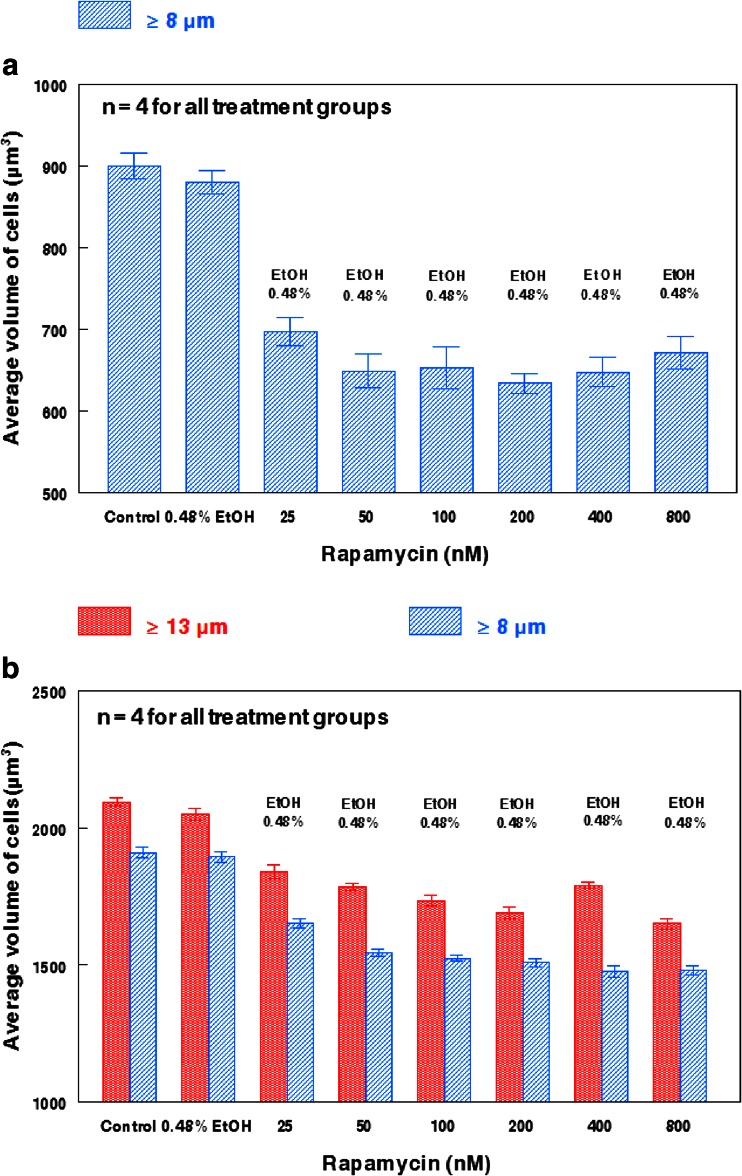
Comparison of rapamycin-induced size reduction between human hematopoietic stem cells and U937 human monocytic leukemia. **a** Average hHSC volume 48 h post-rapamycin administration. All hHSCs were ≥8 μm prior to treatment. **b** Average U937 large and small cell volumes 48 h post-rapamycin administration. Large cells (≥ 13 μm) are denoted with red columns, while small cells (≥ 8 μm) are denoted with blue columns. The effects of 0.48 % EtOH on cell size appear to be minimal. Bars represent the standard error of the mean (SEM) for each individual treatment group.

The effects of rapamycin on the average volume of hHSCs are further highlighted in Fig. 1a. While 0.48 % EtOH appeared to partially reduce hHSC volume (Fig. 1a), the size reduction was minimal, whereas rapamycin from 25 nM to 400 nM produced size reductions from 23 % to 30 % with EtOH concentrations of 0.48 %. Figure 1b shows the effects on average cell volume of leukemia cells treated with rapamycin under conditions identical with the rapamycin treatment of hHSCs shown in Fig. 1a. The EtOH vehicle at 0.48 % had a minimal influence on the cell volume of U937 cells ≥8 μm and ≥13 μm. 25 nM rapamycin reduced the volume of U937 cells ≥13 μm by only 10 %, and increasing the concentration of rapamycin from 50 nM to 800 nM reduced U937 cell volume for cells of the same size range by ~20 %, but no concentration dependence was observed. For U937 cells ≥8 μm, the size reduction was ~22 % with concentrations of rapamycin ranging from 50 nM to 800 nM (Fig. 1b). Therefore, rapamycin concentrations up to 800 nM enhanced or maintained the already large 2 to 2.5-fold size differential between hHSCs and U937 cells.

### Proliferation of U937 human monocytic leukemia and human hematopoietic stem cells in the presence of rapamycin from 0.5 nM to 10,000 nM

**Fig. 2:**
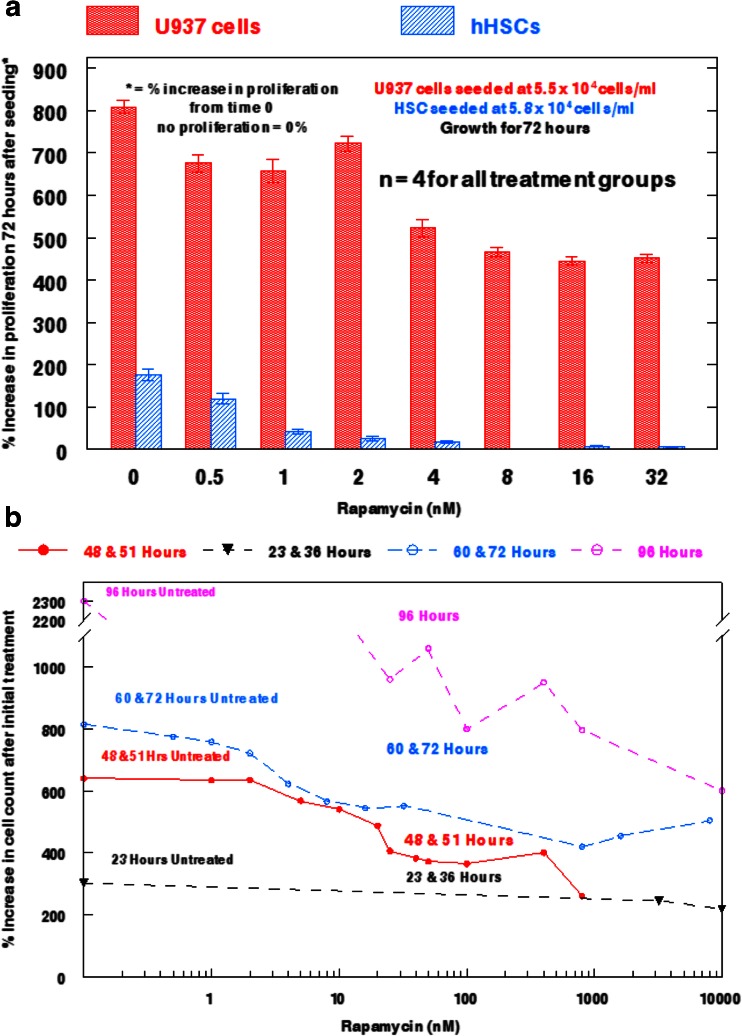
Effects of rapamycin on human hematopoietic stem cell and U937 human monocytic leukemia cell proliferation. **a** Comparison between U937 cells and hHSCs on proliferation rates after exposure to varying concentrations of rapamycin for 72 h. U937 cells are denoted by solid bars, while hHSCs are denoted by hatched bars. Bars represent SEM for each individual treatment group. **b** Time-course study of the effect of rapamycin from 0 to 10,000 nM on U937 cell proliferation. Each line represents the hour post-rapamycin administration at which cell proliferation was observed.

It appeared that U937 cells, but not hHSCs, readily proliferated in a 72 h growth assay in the presence of rapamycin from 0.5 nM to 32 nM (Fig. 2a). Untreated U937 cells increased 8-fold in 72 h, while rapamycin-treated cells exhibited a 5 to 7-fold increase at rapamycin concentrations as high as 32 nM. By contrast, untreated hHSCs doubled in 72 h, but had very low rates of proliferation after treatment with 1 nM to 4 nM rapamycin, and were completely inhibited by rapamycin at 8 nM to 32 nM.

To determine the effects of rapamycin at concentrations above 32 nM, separate U937 cell populations were studied under concentrations of rapamycin ranging from 0.5 nM to 10,000 nM (10 μM) for times of 48 to 51 h, 60 to 72 h, or 96 h (Fig. 2b). The results of this analysis indicated that U937 cells continue to proliferate in the presence of rapamycin at all concentrations from 0.5–100 nM, and remain able to proliferate with slight growth inhibition at concentrations as high as 10 μM. Untreated U937 cells increased 6-fold in 48 to 51 h, 8-fold in 60 to 72 h, and 23-fold in 96 h. In the presence of 10 μM rapamycin, U937 cells were still able to proliferate 6-fold in a 96 h assay. Further, rapamycin did not affect leukemia cell proliferation substantially at 0.5 nM and 1 nM, but did reduce growth in a concentration-dependent fashion from 2 nM to 25 nM. From 25 nM to 10 μM, rapamycin still permitted U937 cell proliferation, allowing treated populations to reach 33–80 % of the proliferation levels obtained with untreated cells. These data suggest that the size and proliferation of hHSCs can be inhibited by rapamycin concentrations that are markedly less inhibitory toward U937 cells.

### Effects of rapamycin-treated U937 cell enlargement following vincristine or cytochalasin B administration

**Fig. 3:**
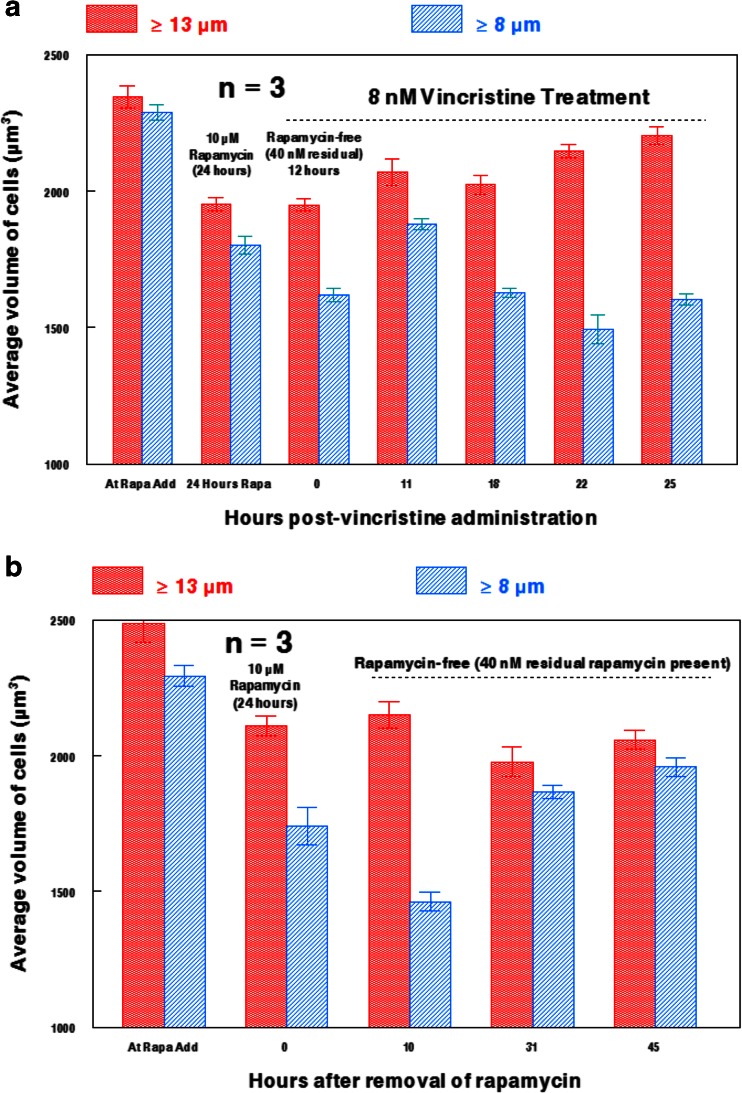
Effects of 8 nM vincristine on rapamycin-treated U937 cells. **a** Effects of 8 nM vincristine for 25 h on U937 cells treated for 24 h with 10 μM rapamycin followed by rapamycin removal prior to vincristine addition. Residual rapamycin estimated at 40 nM after rapamycin removal by dilution. **b** Extent of cell size recovery of U937 cells after removal of 10 μM rapamycin (to a residual 40 nM) with no vincristine added. Large cells (≥ 13 μm) are denoted with red columns, while small cells (≥ 8 μm) are denoted with blue columns. Bars are SEM of three individual cell populations at different time points.

**Fig. 4:**
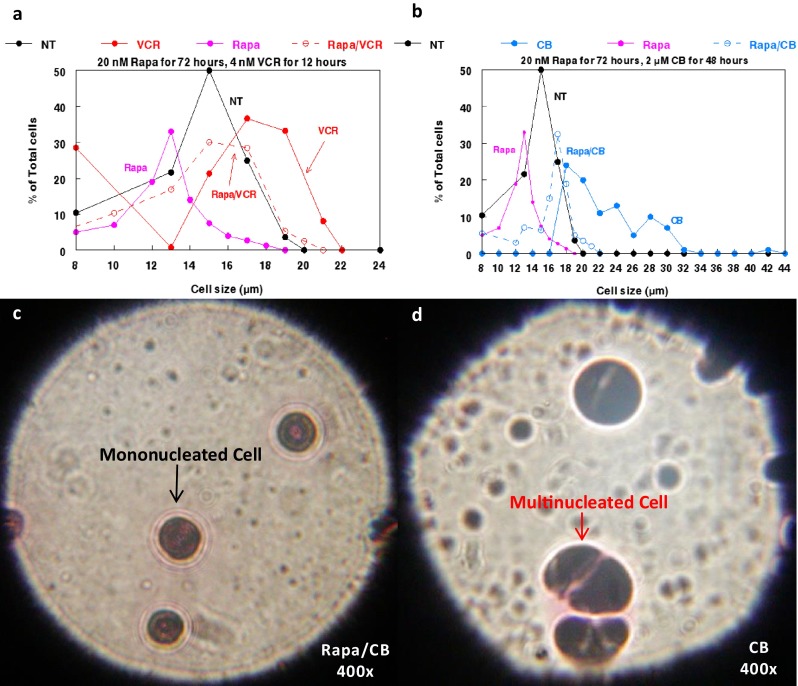
Effects of rapamycin and cytoskeletal-directed agents on cell size of U937 human monocytic leukemia cells. **a** Size distribution of U937 cells following treatment with rapamycin and vincristine alone and in combination. **b** Size distribution of U937 cells following treatment with rapamycin and cytochalasin B alone and in combination. Abbreviations used are as follows: CB (cytochalasin B), NT (not treated), Rapa (rapamycin), and VCR (vincristine). **c** Photomicrograph of U937 cells treated with 2 nM Rapa and 2 μM CB for 48 h. d) Photomicrograph of U937 cells treated with 2 μM CB for 48 h. Photomicrographs were taken at 400× after being Wright stained.

8 nM vincristine appeared to have a notable influence on the size of U937 cells previously treated with 10 μM rapamycin for 24 h (Fig. 3a). The average size of the population appeared to markedly increase after vincristine exposure for 22 or 25 h in the presence of residual rapamycin (the dilution to remove 10 μM left a residual rapamycin concentration of ~40 nM, as indicated in Fig. 3a). This demonstrates that rapamycin-treated leukemia cells can increase in size after exposure to the microtubule-directed agent. By contrast, U937 cells treated for 24 h with 10 μM rapamycin followed by rapamycin removal to 40 nM, but not exposed to vincristine, remained substantially reduced in size for up to 45 h (Fig. 3b). Nevertheless, these data indicate that rapamycin/vincristine treated leukemia cells do not reach the extent of enlargement observed after treatment with only vincristine (Fig. 4a). A similar pattern is observed when U937 cells are exposed to 2 μM cytochalasin B for 48 h, as rapamycin/cytochalasin B treated cells are larger than those only treated with rapamycin, but are not nearly as large as cells treated only with cytochalasin B (Fig. 4b). Interestingly, rapamycin/cytochalasin B treated U937 cells do not exhibit the high rates of multinucleation that is elicited by cytochalasin B-alone treatments, even when rapamycin is administered at 2 nM for 48 h (Figs. 4b and c). This is in accord with the cell size data shown in Fig. 4b, as rapamycin/cytochalasin B treated cells have a substantially smaller size range than cytochalasin B treated cells, indicating that cell enlargement induced by multinucleation is not as prevalent.

### Effects of mTOR inhibitors and cytoskeletal-directed agents alone and in combination against normal and neoplastic hematopoietic cells

**Fig. 5:**
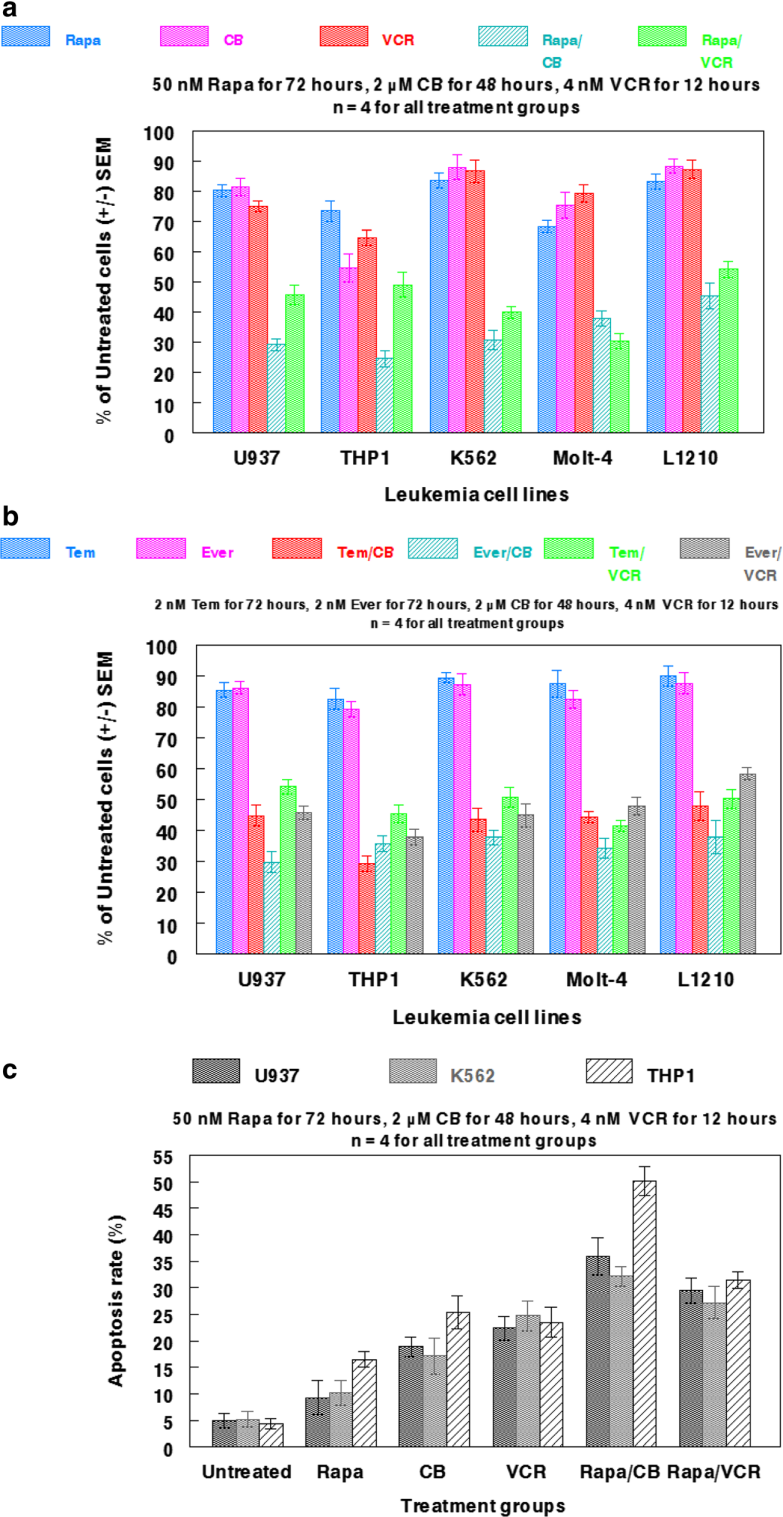
Effects of rapamycin and associated analogs in combination with cytoskeletal-directed agents against normal and neoplastic hematopoietic cells. **a** Multiple leukemia cell lines were treated with 50 nM Rapa for 72 h alone and in combination with either 2 μM CB for 48 h or 4 nM VCR for 12 h. **b** Multiple leukemia cell lines were treated with Rapa analogs everolimus (Ever; 2 nM for 72 h) and temsirolimus (Tem; 2 nM for 72 h) alone and in combination with 2 μM CB for 48 h or 4 nM VCR for 12 h. **c** Annexin V apoptosis assay for U937, K562, and THP1 cells after being treated with rapamycin, cytochalasin B, and vincristine-alone and in combination. Treatment concentrations and durations are indicated in the panel. Bars represent SEM for each individual treatment group.

As illustrated in Fig. 5a, concomitant administration of rapamycin and either cytochalasin B or vincristine perturbed cell viability in all leukemia cell lines examined (U937, THP1, K562, Molt-4, and L1210) much greater than any of the single agent treatments. Of particular note, the percentage of THP1 cells compared to untreated controls dropped from 73.3 % with administration of 50 nM rapamycin to 24.5 % and 48.9 % when combined with either 2 μM cytochalasin B or 4 nM vincristine. By comparison, monotherapy with cytochalasin B or vincristine elicited only 54.6 % and 64.7 %, respectively of the untreated controls. Further, K562 and L1210 were markedly damaged by rapamycin/cytochalasin B or vincristine treatments, despite being notably resistant to all of the single agent treatments, indicative of potentially favorable drug interactions. Concomitant administration of rapamycin/cytochalasin B appeared to potentiate the most damage in all leukemia cell lines, except Molt-4 in which rapamycin/vincristine produced the lowest cell count. Interestingly, the combinatorial effects of rapamycin and cytoskeletal-directed agents were reproduced in all of the leukemic cell lines when rapamycin was replaced with another mTOR inhibitor (either everolimus or temsirolimus; Fig. 5b). These effects were achieved with as little as 2 nM everolimus or temsirolimus, indicative of the potential cytotoxic boost cytoskeletal-directed agents may elicit in combination with mTOR inhibitors. As with rapamycin, it appeared that cytochalasin B potentiated more favorable cytotoxic interaction with everolimus and temsirolimus in comparision to vincristine combinations, thereby producing the lowest cell counts in all leukemia cell lines examined.

The cell viability data were in accord with the annexin V apoptosis assay, as concomitant administration of rapamycin and cytoskeletal-directed agents appeared superior to monotherapy in potentiating apoptotic signaling. As in Fig. 5a, THP1 was particularly sensitive to concomitant chemotherapy, as rapamycin/cytochalasin B produced a 50.1 % apoptosis rate, while rapamycin/vincristine produced a 31.4 % apoptosis rate. In addition, rapamycin/cytochalasin B produced a higher apoptotic rate in all cell lines examined (U937, K562, and THP1) than rapamycin/vincristine, further indicative of unique interactions between mTOR and microfilament inhibition.

### Assessment of drug synergy between rapamycin and cytoskeletal-directed agents

**Fig. 6:**
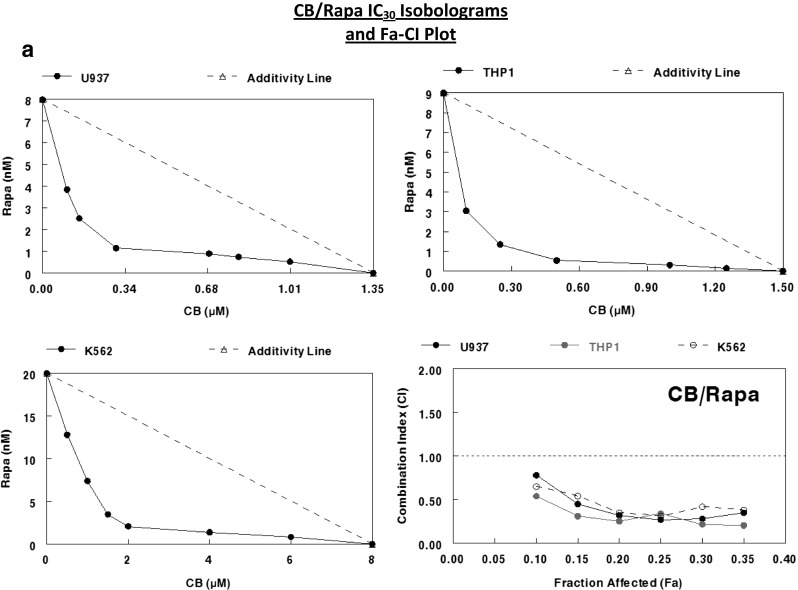

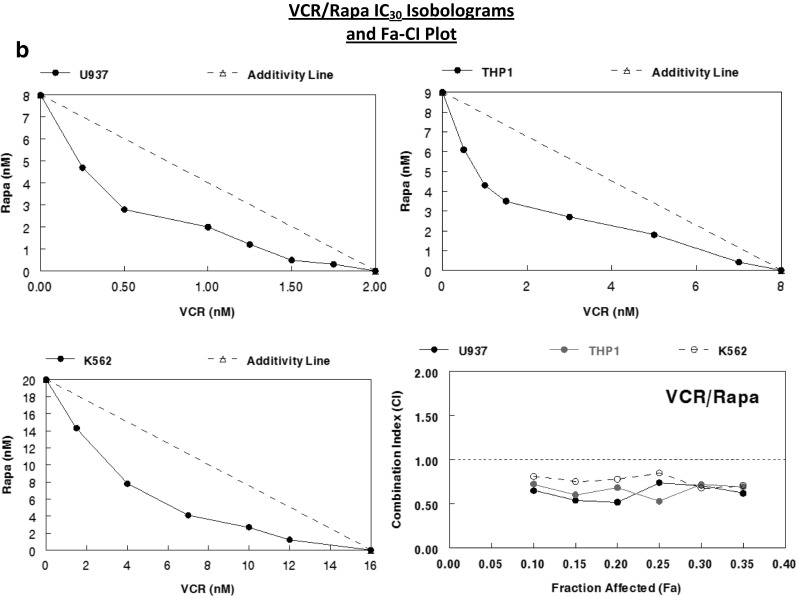
Assessment of drug synergy between rapamycin and cytoskeletal-directed agents against multiple leukemia cell lines after 48 h of continuous exposure through isobolographic analysis and Chou-Talalay method Fa-CI plots. **a** IC_30_ isobolograms and Fa-CI plot for CB/Rapa against U937, THP1, and K562 human leukemia cells. **b** IC_30_ isobolograms and Fa-CI plot for VCR/Rapa against the same cell lines. According to the Chou-Talalay method for the assessment of drug synergy [15], < 0.1 (very strong synergism), 0.1–0.3 (strong synergism), 0.3–0.7 (synergism), 0.7–0.85 (moderate synergism), 0.85–0.9 (slight synergism) are all values of synergism, while 0.9–1.1 may be considered additive or potentially antagonistic, and any values greater than 1.1 are antagonistic.

Rapamycin appeared to synergize with both cytochalasin B and vincristine against U937, THP1, and K562 cells when combined IC_30_ values were plotted to form isobolograms (Fig. 6). However, cytochalasin B interacted more favorably with rapamycin than did vincristine, as the curves underneath the additivity lines were more pronounced, particularly with THP1. Similar results were attained with Chou-Talalay Fa-CI plots (Fig. 6), with many of the rapamycin/cytochalsin B CI points falling in the strong synergism (0.1–0.3) to synergism (0.3–0.7) range, while rapamycin/vincristine CI points often fell in the upper limits of synergism, as well as the moderate synergism (0.7–0.85) range. The potential for increased synergy between rapamycin and cytochalasin B may be the result of the inhibitory effects these agents have on microfilaments, which will be elaborated upon in the discussion.

## Discussion

Rapamycin produced a 24 % reduction in the cell size of hHSCs at 25 nM after 48 h of exposure, and produced a 31 % reduction at 50 nM after the same length of exposure (Table 2). Under these conditions, reduction in cell size for U937 cells was only 12 % for 25 nM and 24 % for 50 nM. This tentatively suggests that rapamycin reduces cell size more in hHSCs than in U937 cells at lower concentrations of rapamycin. Therefore, when both cell populations are compared directly for size reduction after treatment with rapamycin, U937 cells can override the rapamycin treatment under conditions that remain inhibitory for hHSCs. In addition, U937 cells retained the ability to proliferate in the presence of 10 μM rapamycin for 48 h, while hHSC populations were no longer able to proliferate at 8 nM after the same length of exposure (Fig. 2). Further, U937 cells were able to recover from rapamycin exposure, as the percent proliferation was dramatically increased at concentrations ranging from 0.5 nM to 10 μM once the length of exposure was extended to 96 h (Fig. 2b).

These observations suggest that aberrant mTOR signaling in U937 cells may enable such populations to become more resistant to the inhibitory effects of rapamycin compared to normal cell populations. This differential effect of rapamycin on leukemia cells may be related to the overexpression of mTORC1 and potentially mTORC2 in the neoplastic cells, allowing the leukemic cells to proliferate under conditions that reduce the size of hHSCs and drive the normal cells from the cell cycle. Therefore, rapamycin may be able to prevent cell enlargement and proliferation of normal cells under conditions that allow neoplastic cells to enlarge, replicate their nuclei, and potentially multinucleate when treated with microfilament-directed agents that inhibit cytokinesis, although multinucleation was not readily observed in rapamycin/cytochalasin B treated cells (Fig. 4c). This differential effect could protect the small resting normal cells from treatments that damage enlarged, multinucleated, dividing neoplastic cells.

However, it is important to note that rapamycin still produced a substantial reduction in U937 cell size (24 % at 50 nM and 30 % at 800 nM after 48 h of exposure), and that concentrations of rapamycin ≥200 nM inhibited the cell size of U937 cells more than hHSCs, suggesting that mTOR function can be potently inhibited in U937 cells at higher concentrations of rapamycin. Consequently, 10 μM rapamycin-treated U937 cells are unable to substantially recover to normal cell sizes, even 45 h after rapamycin has been removed (note: 40 nM residual rapamycin remained after removal). Although the addition of 8 nM vincristine to rapamycin-treated leukemia cells was enough to nearly restore typical cell sizes observed in populations not treated with rapamycin (Fig. 3b), the cytoskeletal-directed agent was unable to induce the high amount of cell enlargement that is observed with the addition of vincristine as a monotherapy (Fig. 4a).

As assessed through cell viability and drug synergy analyses, mTOR inhibitors (rapamycin, everolimus, and temsirolimus) interact favorably with cytochalasin B and vincristine against all of the leukemic cell lines examined in the present study (U937, THP1, K562, Molt-4, and L1210; Figs. 5 and 6). However, these data also suggest that cytochalasin B may elicit more synergistic potential with mTOR inhibitors than does vincristine, as most, but not all, neoplastic cell populations were inhibited greater with mTOR inhibitor/cytochalasin B administration (Fig. 5). In addition, rapamycin/cytochalasin B elicited more prominent synergistic interactions than rapamycin/vincristine against U937, THP1, and K562 (Fig. 6). The efficacy of rapamycin/vincristine combinations has been previously described, as it has been shown than rapamycin can markedly sensitize vincristine resistant cells to the microtubule-directed agent [16]. Further, the concomitant administration of mTOR inhibitors/vincristine has been demonstrated to be much more effective against multiple murine xenograft models of malignancy than the respective standard agents used alone at their maximum tolerated doses (MTDs) [17, 18]. However, the present study is the first to demonstrate the potential utility of cytochalasin B and possibly other microfilament-directed agents in combination with mTOR inhibitors, and future preclinical studies should be directed towards a more extensive examination of this unique antineoplastic strategy.

Although the underlying mechanistic interactions that promote rapamycin/cytochalasin B drug synergy are not fully elucidated in the present study, it is known that mTORC2 is involved in promoting actin polymerization, thereby enabling the formation of filamentous (F)-actin stress fibers [19–21]. In addition, mTORC2 regulates the actin cytoskeleton through its stimulation of paxillin, RhoA, Rac1, Cdc42, and protein kinase C α (PKCα) [22]. While low concentrations of rapamycin preferentially inhibit mTORC1 rather than mTORC2, the compound begins to inhibit mTORC2 under prolonged exposure [23–25]. Disrupting the formation of F-actin has deleterious effects on the cytoskeletal integrity of cells [26, 27], suggesting that rapamycin should be able to act in concert with cytoskeletal-disrupting antineoplastic agents, and enhance the combined cytotoxicities of both classes of agents. Further, since inhibiting the formation of viable microfilaments through the administration of cytochalasin B dramatically increases the ultrasonic sensitivity of multiple leukemia cells, concomitant administration of rapamycin and cytochalasin B is likely to induce synergistic ultrasonic sensitization. Indeed, we have performed preliminary experiments that indicate rapamycin, everolimus, and temsirolimus administered at concentrations as low as 2 nM potentiate the ultrasonic sensitivity of human leukemia cells, a notion that will be elaborated upon in a subsequent study.

It should be noted that while cytoskeletal-directed agents enhanced the cytotoxicity of mTOR inhibitors, as well as enlarged U937 cells treated with rapamycin, concomitant administration of rapamycin and cytoskeletal-directed agents may preclude significant increases in neoplastic cell size, as is observed when either cytochalasin B or vincristine are administered as single agents (Fig. 4a; 12, 27]). Nevertheless, we have also determined that the mechanism by which cytoskeletal-directed agents induce cell enlargement is vital for their ultimate potentiation of ultrasonic sensitivity, with agents that inhibit polymerization eliciting a much greater effect than those that enhance and stabilize formed cytoskeletal polymers [27, 28]. Therefore, concomitant administration of cytochalasin B and/or vincristine with mTOR inhibitors will likely potentiate marked ultrasonic sensitization.

The present study suggests that rapamycin and cytoskeletal-directed agents may potentiate clinically applicable drug synergy, while concurrently inducing a slight, but noticeable preferential increase of malignant cell size in the presence of their normal counterparts. As indicated by the study, rapamycin should be administered first, effectively deactivating the proliferation potential of normal blood cells. Then, cell enlarging cytoskeletal-directed agents that act during mitotic events can be applied to preferentially affect actively proliferating neoplastic cells. Such concomitant chemotherapy may increase the substantial size difference between malignant cells and those of normal histology; the exact feature by which SDT preferentially damages neoplastic tissue. Regardless of their potential use as sonosensitizers, rapamycin, everolimus, and temsirolimus all appear to demonstrate enhanced cytotoxicity when used in combination with cytochalasin B or vincristine. Further characterizing the potential antineoplastic applications of mTOR inhibitors and cytoskeletal-directed agents using in vivo preclinical mammalian models of malignancy is therefore warranted.
